# Association between alcohol consumption and osteoarthritis prevalence in Korea as assessed by the alcohol use disorders identification test (AUDIT): a cross-sectional study

**DOI:** 10.1186/s12889-020-8326-4

**Published:** 2020-02-13

**Authors:** Ah. Hyun Kang, Me-riong Kim, Joon-Shik Shin, Jinho Lee, Yoon Jae Lee, Yeoncheol Park, Dongwoo Nam, Eun-Jung Kim, In-Hyuk Ha

**Affiliations:** 1 Incheon Jaseng Hospital of Korean Medicine, Incheon, Republic of Korea; 2 Jaseng Hospital of Korean Medicine, Seoul, Republic of Korea; 3 Jaseng Spine and Joint Research Institute, Jaseng Medical Foundation, Seoul, Republic of Korea; 4 Department of Acupuncture & Moxibustion, Kyung Hee University Hospital at Gangdong, Seoul, Republic of Korea; 5 Department of Acupuncture & Moxibustion, College of Korean Medicine, Kyung Hee University, Seoul, Republic of Korea; 6 Department of Acupuncture & Moxibustion, College of Korean Medicine, Dongguk University, Gyeongju, Republic of Korea

**Keywords:** Alcohol, Osteoarthritis, Epidemiology, Cross-sectional studies, Health surveys

## Abstract

**Background:**

Osteoarthritis (OA) holds significance as a highly prevalent disorder in elderly populations. Various studies have been conducted on the association between alcohol consumption and OA, but the results have often been conflicting. The aim of this study was to investigate the relationship between alcohol consumption and OA in a large-scale sample representative of the Korean population.

**Methods:**

Among the 25,534 participants surveyed in the fifth Korean National Health and Nutrition Examination Survey (2010–2012), 7165 individuals aged ≥50 who responded to drinking-related items were analyzed. The Alcohol Use Disorders Identification Test (AUDIT) grade was calculated, and radiologic examination analysis included the Kellgren-Lawrence (KL) grade of the lumbar spine, hip, and knee joints. Logistic regression analysis was performed to evaluate the association between AUDIT grades and OA through estimation of odds ratios (ORs).

**Results:**

In crude analyses, OA (KL grade ≥ 2) of the lumbar spine and knee was more prevalent towards Zone I, but following adjustment, knee OA prevalence significantly increased in Zone III and IV compared to Zone I (Zone III: OR 1.464, 95% confidence interval (CI) 1.027–2.088; Zone IV: OR 1.543, 95% CI 1.028–2.317, respectively). Meanwhile, adjusted hip and lumbar OA values showed positive associations towards Zone IV, but did not reach statistical significance. Additional analyses of the association between alcohol consumption and pain severity of knee OA patients were nonsignificant.

**Conclusions:**

These results imply that radiological knee OA, rather than symptomatic knee OA, is associated with alcohol consumption.

## Background

Osteoarthritis (OA) is a chronic degenerative joint disorder characterized by synovial inflammation, osteophyte formation, and loss of cartilage with cartilage and bone sclerosis, leading to dysfunction, stiffness and pain of the joint [1]. OA may affect all synovial joints but is most common in weight-bearing joints such as the knee, hip, foot and spine, and joints of the hand. Its prevalence is high, and symptomatic OA is estimated to affect 1 in 8 Americans (total 27–31 million) [2, 3], with global estimates for knee OA reaching 250 million [4].

Between 1990 and 2010, OA-related disability in Britain increased 16% [4], and Woolf et al. predicted that OA would become the fourth leading cause of disability by 2020 [5]. Seventeen million of the British population are expected to suffer from OA by 2030 [6], while a study by Johnson et al. put U.S. estimates of OA at 67 million by 2030, purporting that OA would be a major cause of medical expenditure [7]. OA-related expenses have been associated with substantial economic costs equaling approximately 1~2.5% of gross domestic product (GDP) of developed countries [8, 9].

Current evidence indicates that the cause of OA is multifactorial, and research has been conducted on major risk factors including age, sex, obesity, and geographic and genetic factors [10]. Alcohol consumption has also been proposed as a potential risk factor for OA. In an in vivo study by Kc et al. [11], chronic alcoholic intake in mice not only led to direct toxicity, but various indirect changes at molecular level including metabolite production, oxidative stress, inflammation, and immune reactions associated with OA. A prospective study on alcohol drinkers aged 45 and over in Australia also found that alcohol consumption cessation was significantly related with the frequency with which they experienced treatment for OA [12].

Meanwhile, a cross-sectional study by Andrianakos et al. on prevalence of rheumatic diseases in Greece failed to find a significant association between alcohol consumption and OA [13], and a study on risk factors associated with total hip replacement due to OA was likewise unable to identify a significant relationship between alcohol consumption and OA [14].

However, most studies assessing alcohol consumption as a potential risk factor for OA were limited in that (1) alcohol consumption was not analyzed as the main variable [13, 14], (2) diagnoses were not made with consideration of radiologic findings [13, 14], and (3) OA prevalence of only single joints or in generalization was assessed [12–14]. In addition, the existing literature shows considerable discordance between clinical and radiographic knee OA, and a wide variation in the degree that X-ray findings and symptoms are related. Therefore, to comprehensively investigate the association between alcohol consumption and OA prevalence, a large-scale study based on radiological diagnosis of OA in the knee, hip joint, and spine, which are the major regions affected by OA, with adjustment for covariates and additional analysis for OA-related pain symptoms was conducted.

## Methods

### Study population and sampling

The present study investigated the association between alcohol consumption and OA prevalence and OA-related pain in Koreans using data from the Korean National Health and Nutrition Examination Survey (KNHANES). KNHANES is a national sample survey conducted by the Korea Centers for Disease Control and Prevention (KCDC) under the Korean Ministry of Health and Welfare in South Korea. KNHANES is conducted to evaluate the health and nutritional status for the Korean people. The Korean population residing in Korea with the exception of foreigners, military service personnel, and nursing home and correctional facility residents participated in KNHANES. KNHANES data can be accessed and downloaded from the KNHANES website (https://knhanes.cdc.go.kr/knhanes/index.do). Data were obtained from the fifth KNHANES 2010–2012 (KNHANES V).

Twenty households are selected out of 192 regions each year, and 10,000 individuals aged ≥1 year are the target population for KNHANES. The KNHANES uses a complex, multi-stage probability sample design. The sample collectively represents the total non-institutionalized civilian population of Korea. The survey components are divided into 3 parts; Health Examination, Health Interview, and Nutrition Survey, and the survey and examination items and methods used are determined by KCDC and related academic societies to better monitor trends in risk factors for health and prevalence of major chronic diseases, and provide data for development and evaluation of South Korean health policy and programs. The health interview and examination are conducted by trained staff, including physicians, technicians and interviewers, by means of a mobile examination center, and follow-ups are conducted through dieticians’ visits to the homes of participants [15].

**Fig. 1:**
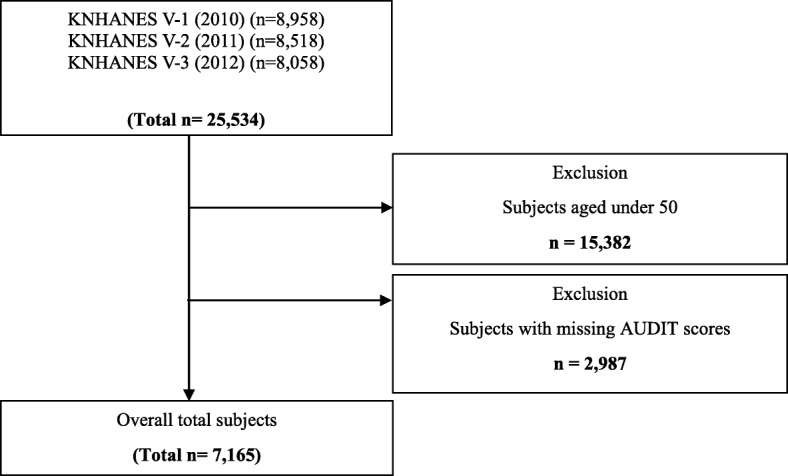
Flow diagram of study

Among the 25,534 participants surveyed in KNHANES V, 7165 individuals aged 50 years or over who responded to drinking-related items were analyzed. Therefore, subjects aged under 50 years (*n* = 15,382), and subjects with missing Alcohol Use Disorders Identification Test (AUDIT) scores (*n* = 2987) were excluded. Ultimately, the analyses were performed using data of 7165 subjects, of which the subject inclusion and exclusion process is described in more detail in Fig. 1.

### OA

OA screening was conducted by radiologists using X-rays in accordance with the “Professional Surveyor Education and Quality control for OA Examination.” OA examination and readings were performed by two radiologists using the Kellgren-Lawrence (KL) grading system. If the grades are discrepant by more than 2 grades, those digital data were read by another radiologist.

The KL grading classification was originally described with AP knee radiographs. Each radiograph was assigned a grade or 0–4, which correlated to increasing severity of OA (Grade 0 signifying no presence of OA, and Grade 4 severe OA). Additionally, the KL grading system provides detailed radiographic descriptions of OA as follows [16]:

- 0: No joint space narrowing or reactive changes
- 1: Doubtful joint space narrowing, possible osteophytic lipping
- 2: Definite osteophytes, possible joint space narrowing
- 3: Moderate osteophytes, definite joint space narrowing, some sclerosis, possible bone-end deformity
- 4: Large osteophytes, marked joint space narrowing, severe sclerosis, definite bone ends deformity

### OA of the knee joint

KL grades of ≥2 as assessed by digital X-ray images of the knee joint were considered radiological OA of the knee joint [16].

### OA of the hip joint

KL grades of ≥2 as assessed by digital X-ray images of the hip joint were regarded radiological OA of the hip joint [16].

### OA of the lumbar spine

OA of the lumbar spine was evaluated through digital X-ray images of the lumbar spine in accordance with KL grades as follows: 0 = normal, 1 = definite osteophyte, and 2 = intervertebral disk space narrowing, bone sclerosis, and large osteophytes. KL grades were assessed by digital X-ray images of the lumbar spine in consideration of radiological OA of the lumbar spine [16].

### Knee pain

Self-reported knee pain was assessed as a survey item included in the Health Interview, and pain severity was measured using the numeric rating scale (NRS). Participants who answered “Yes” to the question “Have you experienced knee pain for 30 days or more over the past three months?” were asked “What is your average knee pain (irrespective of whether you take medication or not)?” Participants indicated their answers using NRS values from 0 to 10 (0 indicative of no pain, and 10, worst pain imaginable).

### Alcohol consumption

AUDIT developed by the World Health Organization (WHO) was employed to assess alcohol consumption. AUDIT is a concise measurement tool that evaluates excessive drinking, and was developed to examine the potential contribution of alcohol to development of various disorders [17]. The test is comprised of 10 items which measure the level of alcohol consumption and dependence, and the total sum of scores adds up to 40, with scores of ≥8 indicative of hazardous and harmful alcohol use. Of the 10 items, questions 1–3 measure hazardous alcohol use, and investigate the quantity and frequency of drinking and heavy drinking. Questions 4–6 pertain to alcohol dependency, and inquire into impaired control over drinking, increased salience of drinking, and morning drinking. The remaining 4 items (questions 7–10) ask about resulting recent and lifetime problems from harmful alcohol use, such as guilt after drinking, blackouts, alcohol-related injuries, and whether others are concerned about the respondent’s drinking.

Drinking habits as assessed by AUDIT were classified into 4 zones by level of intervention and risk. Zone I indicates non-drinking, abstinence or low risk drinking with an AUDIT score of ≤7 and intervention level of alcohol education. An AUDIT score of ≥8 is indicative of high risk drinking [18]: Zone II indicates alcoholic use beyond low-risk guidelines with an AUDIT score of 8–15 [19]; Zone III indicates an AUDIT score of 16–19 and intervention level covers brief counseling and continued monitoring in addition to simple advice; and Zone IV indicates an AUDIT score of ≥20 which suggests that the respondent is in need of professional treatment for alcohol dependency. Brief counseling may vary from simple 5-min advice to the aim of lowering dangerous drinking, to multiple counseling sessions in more serious cases. Brief counseling for Zone III respondents is characterized by low intensity and short duration [20].

### Covariates

The sociodemographic characteristics of participants investigated in the survey included age, sex, education, income, occupation, marital status, and area of residence. Health-related characteristics included such factors as smoking, body mass index (BMI), and regular exercise.

Education level was categorized into 4 levels: elementary school graduate or lower, middle school graduate, high school graduate, and college graduate or higher. Income level was classified into quartiles by individual income. Occupation was classified into the following 7 categories with the exclusion of military service: (1) managing, administrative and professional positions, (2) office workers, (3) service and sales industry workers, (4) agriculture and fishery workers, (5) engineering and equipment and machinery operation and assembly workers, (6) simple laborers, and (7) unemployment (which included housewives and students). BMI (kg/m^2^) was further classified into underweight, normal and overweight according to the most commonly used definitions established by the WHO as < 18.5, 18.5 ≤ BMI < 25, and ≥ 25, respectively. Smoking status was trichotomized into (1) former smoker if the person had smoked ≥5 packs of cigarettes in the past and did not smoke at present, (2) current smoker if the person had smoked ≥5 packs in the past and continued to smoke, and (3) non-smoker if they indicated otherwise. Regular exercise was defined as engaging in (1) ≥20 min of rigorous regular exercise more strenuous than usual (e.g., running, climbing, cycling) for ≥3 days during the past week, (2) ≥30 min of regular exercise a little more challenging than usual (e.g., swimming, doubles tennis, volleyball) for ≥5 days during the past week, or (3) ≥30 min of walking for ≥5 days during the past week.

### Statistical methods

KNHANES is a nationwide sample survey that employs stratified cluster sampling and weighted values. Stratification, clustering, and weighting were accordingly included as complex sample design elements in this complex sample data analysis. Statistical analyses were performed with SAS version 9.4 package (SAS Institute Inc., Cary, NC, USA), with *p* < 0.05 regarded to be statistically significant. Continuous variables are presented as mean ± standard deviation (SD), and nominal variables as frequency and percentiles (%). Difference in participant characteristics by drinking and OA was assessed using Rao-Scott Chi-Square test, or ANOVA in analysis of ≥3 groups. Logistic regression analysis for complex sample design was conducted to evaluate the association between AUDIT scores and OA after adjusting for covariates. Odds ratios (ORs) and 95% confidence intervals (CIs) were calculated with age and BMI as continuous variables, and other variables as nominal variables in adjustment for covariates.

## Results

### Participant demographics by alcohol consumption

Table 1 presents the associations between sociodemographic characteristics and alcohol consumption. Following increase of AUDIT scores, participant age decreased and the ratio of male participants significantly increased (*p* < .0001). While the rate of unemployed individuals was high in Zone I, most occupations showed higher percentages in Zones II and III. The percentage of former and present smokers was higher than that of non-smokers following increase in AUDIT scores (*p* < .0001).

**Table 1:** Demographic characteristics of KNHANES surveyees aged ≥50 by AUDIT scores

Factors / Subgroup	AUDIT score^a^								
	Zone I		Zone II		Zone III		Zone IV		
Total	5220		1187		369		389		
	*N*	%	*N*	%	*N*	%	*N*	%	*p* value
Age (years)^**b**^	62.39 ± 0.18		59.33 ± 0.28		57.82 ± 0.38		57.43 ± 0.38		
Sex									
Male	1985	38.0	973	82.0	346	93.8	366	94.1	< 0.001^*^
Female	3235	62.0	214	18.0	23	6.2	23	5.9	
Household income									
Lower quartile	1605	31.1	290	24.8	78	21.2	78	20.3	< 0.001^*^
Middle-lower quartile	1327	25.7	309	26.4	81	22.1	102	26.6	
Middle-upper quartile	1111	21.5	259	22.1	85	23.2	98	25.5	
Upper quartile	1117	21.7	313	26.7	123	33.5	106	27.6	
Education									
Elementary school graduate or lower	2475	47.4	392	33.1	113	30.6	114	29.5	< 0.001^*^
Middle school graduate or lower	881	16.9	248	21.0	61	16.5	84	21.7	
High school graduate or lower	1217	23.4	366	31.0	129	35.0	127	32.8	
College graduate or higher	640	12.3	176	14.9	66	17.9	62	16.0	
Occupation									
Managing, administrative and professional positions	280	5.4	96	8.2	36	9.8	32	8.3	< 0.001^*^
Office work	119	2.3	50	4.2	28	7.6	25	6.5	
Service and sales industry	541	10.4	140	11.9	36	9.8	47	12.1	
Agriculture and fishery	686	13.2	191	16.2	72	19.6	70	18.1	
Engineering and equipment and machinery operation and assembly	312	5.9	192	16.3	68	18.5	76	19.6	
Simple labor	624	12.0	134	11.4	34	9.2	39	10.1	
Unemployment (including housewives and students)	2646	50.8	375	31.8	94	25.5	98	25.3	
Marital status									
Married	5176	99.2	1176	99.2	365	98.9	383	98.7	0.138
Single	42	0.8	10	0.8	4	1.1	5	1.3	
Urban-rural gradient									
Urban area	3840	73.6	883	74.4	270	73.2	270	69.4	0.422
Rural area	1380	26.4	304	25.6	99	26.8	119	30.6	
Smoking status									
Non-smoker	3356	64.3	315	26.5	63	17.1	56	14.4	< 0.001^*^
Former smoker	1229	23.6	464	39.1	164	44.4	162	41.7	
Current smoker	633	12.1	408	34.4	142	38.5	171	43.9	
Body mass index (kg/m^2^)									
mean ± SD	24.00 ± 0.06		24.22 ± 0.10		23.83 ± 0.17		24.08 ± 0.19		
Underweight	141	2.7	22	1.9	11	3.0	8	2.1	0.862
Normal	3296	63.3	721	61.0	234	63.6	238	61.3	
Overweight	1769	34.0	439	37.1	123	33.4	142	36.6	
Regular exercise									
Yes	458	8.8	128	10.8	38	10.3	32	8.2	
No	4753	91.2	1057	89.2	330	89.7	357	91.8	0.126

*KNHANES* Korean National Health and Nutrition Examination Survey; *AUDIT* Alcohol Use Disorders Identification Test;

^*^Statistical significance was set as *p* < 0.05

^a^ AUDIT scores are calculated as the sum of 10 items, and were categorized into Zone I (0–7), Zone II (8–15), Zone III (16–19), and Zone IV (20+)

^**b**^ Continuous variables are presented as mean ± standard deviation (SD)

### Distribution of OA (KL grade) by alcohol consumption

Table 2 presents the associations between the KL grade of the hip joint, knee, and lumbar spine and AUDIT scores, respectively. OA prevalence in each joint as assessed by KL grades was distributed differently by AUDIT zone. Non OA groups (KL grade 0) decreased, and OA groups (KL grades of ≥2) increased towards Zone I from Zone IV.

**Table 2:** Kellgren-Lawrence (KL) grade of the hip, knee, and lumbar spine by AUDIT scores in KNHANES surveyees

KL grade	AUDIT score^**a**^								*p* value
	Zone I		Zone II		Zone III		Zone IV		
	*N*	%	*N*	%	*N*	%	*N*	%	
Hip joint									
0 (normal)	4235	82.3	929	79.1	279	76.8	303	78.5	< 0.001^*^
1 (suggestive of OA)	870	16.9	230	19.6	83	22.9	78	20.2	
2 (non-severe OA)	33	0.6	13	1.1	0	0.0	4	1.0	
3 (severe OA)	12	0.2	2	0.2	1	0.3	1	0.3	
Knee joint									
0 (normal)	2065	40.0	547	46.6	172	47.4	189	49.0	< 0.001^*^
1 (suggestive of OA)	1220	23.7	342	29.2	102	28.1	106	27.5	
2 (mild OA)	729	14.2	150	12.8	56	15.4	59	15.3	
3 (moderate OA)	775	15.1	92	7.9	29	8.0	23	5.9	
4 (severe OA)	362	7.0	41	3.5	4	1.1	9	2.3	
Lumbar spine									
0 (normal)	1100	21.8	244	21.2	72	20.6	68	18.3	< 0.001^*^
1 (suggestive of OA)	2228	44.1	598	52.1	188	53.7	199	53.6	
2 (OA)	1718	34.1	307	26.7	90	25.7	104	28.1	

*KL* Kellgren-Lawrence; *AUDIT* Alcohol Use Disorders Identification Test; *KNHANES* Korean National Health and Nutrition Examination Survey

^*^Statistical significance was set as *p* < 0.05

^a^ AUDIT scores are calculated as the sum of 10 items, and were categorized into Zone I (0–7), Zone II (8–15), Zone III (16–19), and Zone IV (20+)

### Association between alcohol consumption and OA (KL grade)

Table 3 presents the association between alcohol consumption and risk of OA, adjusted for covariates. OR values of each joint were calculated through comparison of KL grades ≥2 and grade 0 (reference), respectively.

**Table 3:** Associations between AUDIT scores and OA prevalence of the hip joint, knee, and lumbar spine in KNHANES surveyees

Joint location	AUDIT score ^a^	OA prevalence		Crude			*p* value	Age- and sex-adjusted			*p* value	Fully-adjusted^b^			*p* value
		No	Yes	OR	95% CI			OR	95% CI			OR	95% CI		
Hip OA	Zone I	4235	45	1.00				1.00				1.00			
	Zone II	929	15	1.73	0.85	3.52	0.129	1.44	0.67	3.12	0.349	1.28	0.57	2.92	0.548
	Zone III	279	1	0.49	0.06	3.69	0.484	0.39	0.05	3.01	0.368	0.41	0.05	3.21	0.398
	Zone IV	303	5	1.52	0.54	4.31	0.426	1.27	0.42	3.83	0.676	1.24	0.40	3.88	0.712
Knee OA	Zone I	2065	1866	1.00				1.00				1.00			
	Zone II	547	283	0.51	0.42	0.61	**< 0.001**	1.07	0.86	1.32	0.555	1.00	0.80	1.26	0.978
	Zone III	172	89	0.50	0.37	0.68	**< 0.001**	1.48	1.03	2.12	**0.032**	1.46	1.03	2.09	**0.036**
	Zone IV	189	91	0.51	0.37	0.71	**< 0.001**	1.61	1.10	2.37	**0.015**	1.54	1.03	2.32	**0.036**
Lumbar spine OA	Zone I	1100	1718	1.00				1.00				1.00			
	Zone II	244	307	0.68	0.55	0.85	**0.001**	0.93	0.71	1.22	0.596	0.87	0.66	1.16	0.355
	Zone III	72	90	0.62	0.42	0.91	**0.015**	1.00	0.65	1.53	0.992	0.98	0.61	1.57	0.923
	Zone IV	68	104	0.78	0.53	1.16	0.222	1.48	0.91	2.40	0.110	1.29	0.77	2.16	0.335

*AUDIT* Alcohol Use Disorders Identification Test; *OA* Osteoarthritis; *OR* Odds ratio; *CI* Confidence interval

^*^Statistical significance was set as *p* < 0.05

^a^ AUDIT scores are calculated as the sum of 10 items, and were categorized into Zone I (0–7), Zone II (8–15), Zone III (16–19), and Zone IV (20+)

^b^ Fully-adjusted: adjusted for age, sex, income level, educational level, occupation, marital status, residential area, smoking status, body mass index, and physical activity

In crude analysis, OA of the knee implied a negative relationship in AUDIT Zones II, III, and IV, and OA of the lumbar spine, a negative relationship in AUDIT Zones II, and III. However, following age- and sex-adjustments and full-adjustments, significant positive associations were shown for knee OA in Zones III, and IV, indicating increasing risk of OA with higher AUDIT scores (Zone III: OR 1.464, 95% CI 1.027–2.088; Zone IV: OR 1.543, 95% CI 1.028–2.317). While age- and sex-adjusted, and fully-adjusted values for OA of the hip and lumbar spine likewise displayed positive associations with Zone IV, they did not reach statistical significance.

### Association between alcohol consumption and severity of pain in knee OA patients

Significant associations between OA and alcohol consumption were observed only for the knee joint. Additional analyses were conducted to examine potential associations between alcohol consumption and pain severity in knee OA patients, and the results are given in Table 4. Model 1 was constructed to compare presence of pain (NRS 1–10) with no pain (NRS 0). The association was not significant, even after sex- and age-adjustments and full adjustments. Model 2 was constructed to compare moderate and severe pain (NRS 4–10), with mild pain of NRS ≤3 or less. Similarly, the association was nonsignificant. Models 3 and 4 show comparisons of moderate or higher pain (NRS 4–10), and severe or higher pain (NRS 7–10), with no pain (NRS 0), respectively, the results of which were also nonsignificant.

**Table 4:** Association between AUDIT scores and pain severity of the knee joint in KNHANES surveyees

Models	AUDIT score^e^	N (case)	Crude				Age- and sex-adjusted				Fully-adjusted^**f**^			
			OR	95% CI		*p* value	OR	95% CI		*p* value	OR	95% CI		*p* value
Model 1^a^	Zone I	1860 (708)	1.00				1.00				1.00			
	Zone II	283 (78)	0.59	0.42	0.82	0.002^*^	1.08	0.76	1.55	0.671	1.07	0.72	1.57	0.748
	Zone III	89 (23)	0.52	0.28	0.94	0.031^*^	1.26	0.67	2.39	0.477	1.23	0.61	2.46	0.560
	Zone IV	91 (19)	0.32	0.17	0.60	0.000^*^	0.90	0.47	1.71	0.740	0.86	0.43	1.73	0.678
Model 2^b^	Zone I	1860 (622)	1.00				1.00				1.00			
	Zone II	283 (65)	0.54	0.39	0.76	< 0.001^*^	0.99	0.69	1.41	0.946	0.94	0.64	1.38	0.768
	Zone III	89 (19)	0.49	0.26	0.95	0.036^*^	1.21	0.60	2.41	0.597	1.21	0.57	2.56	0.621
	Zone IV	91 (17)	0.32	0.17	0.60	0.001^*^	0.88	0.44	1.75	0.708	0.85	0.40	1.78	0.656
Model 3^c^	Zone I	1774 (622)	1.00				1.00				1.00			
	Zone II	270 (65)	0.54	0.38	0.77	0.001^*^	1.01	0.70	1.45	0.963	0.97	0.65	1.45	0.880
	Zone III	85 (19)	0.49	0.25	0.94	0.033^*^	1.23	0.61	2.48	0.565	1.21	0.56	2.60	0.631
	Zone IV	89 (17)	0.31	0.16	0.59	0.000^*^	0.89	0.45	1.79	0.745	0.86	0.41	1.84	0.704
Model 4^d^	Zone I	1516 (364)	1.00				1.00				1.00			
	Zone II	234 (29)	0.41	0.25	0.67	< 0.001^*^	0.89	0.53	1.48	0.641	0.83	0.48	1.44	0.507
	Zone III	77 (11)	0.46	0.21	0.97	0.041^*^	1.49	0.67	3.31	0.333	1.25	0.52	3.02	0.615
	Zone IV	79 (7)	0.20	0.08	0.48	< 0.001^*^	0.79	0.31	1.98	0.609	0.74	0.28	1.92	0.531

*AUDIT* Alcohol Use Disorders Identification Test; *KNHANES* Korean National Health and Nutrition Examination Survey

^*^Statistical significance was set as *p* < 0.05

^a^ Model 1: Odds ratios for NRS 1–10 was assessed against a reference of NRS 0 or no pain

^b^ Model 2: Odds ratios for NRS 4–10 was assessed against a reference of NRS 0–3 or no pain

^c^ Model 3: Odds ratios for NRS 4–10 was assessed against a reference of NRS 0 or no pain

^d^ Model 4: Odds ratios for NRS 7–10 was assessed against a reference of NRS 0 or no pain

^e^ AUDIT scores are calculated as the sum of 10 items, and were categorized into Zone I (0–7), Zone II (8–15), Zone III (16–19), and Zone IV (20+)

^**f**^ Fully-adjusted: adjusted for age, sex, income level, educational level, occupation, marital status, residential area, smoking status, body mass index, and physical activity

## Discussion

This study analyzed KNHANES V data (2010–2012) to investigate the association between alcohol consumption and OA of various joints in Koreans aged ≥50. In crude analysis, prevalence of non-OA decreased and OA of KL grades ≥2 increased towards Zone I from Zone IV, while adjusted results showed that Zones III and IV (higher scores indicate higher alcohol dependency) displayed significantly higher prevalence of knee OA compared to Zone I. Meanwhile, adjusted results of hip joint and lumbar spine OA showed positive, but nonsignificant associations with Zone IV. Further analysis of the association between alcohol consumption and pain severity of knee OA patients did not reach statistical significance.

The difference in direction of association between alcohol consumption and OA in crude and adjusted analyses, with adjustment for various confounding factors such as sex and age, is conjectured to be the result of a higher percentage of females, who are more prone to OA, in Zone I. Also, while adjusted results resulted in significantly higher prevalence of knee OA in Zones III and IV compared to Zone I, hip joint and lumbar spine OA showed positive associations with Zone IV, but did not reach statistical significance. However, taking into consideration the low prevalence of hip joint and lumbar spine OA, the lack of statistical power due to small sample size may explain the nonsignificance of these results.

A number of studies have been published on alcohol consumption as a possible risk factor for OA. Most clinical studies, however, have failed to find significant associations, such as in a Finnish cohort study with an observation period of 22 years [21] and the Nurses’ Health Study [14], a cross-sectional study in American women, where the association between alcohol intake and hip joint OA did not show significance, and the association between alcohol consumption and plasma high-sensitivity C-reactive protein (hsCRP) levels was also shown to be nonsignificant in a cross-sectional study on early radiological knee OA patients [22]. On the other hand, a handful of studies including the present study suggest the possibility of alcohol intake as a risk factor for OA. In an in vivo study by Kc et al. [11], chronic intake of alcohol led to pathological changes similar to OA. Chronic and excessive intake of alcohol has been associated with high levels of inflammatory mediator circulation [23], and inflammation has been found to be a predictive factor of structural damage in hand joint OA [24]. Taken together, excessive alcohol consumption is suggested to induce inflammation at local sites, which contributes to the pathogenesis of OA. However, moderate alcohol intake has been reported to be associated with anti-inflammatory effects [25], preventing definite conclusions being drawn on the subject.

In the current study, adjusted analysis of alcohol intake with joint pain severity did not yield significant associations. Whether alcohol relieves or aggravates pain remains a matter of controversy. Alcohol is a psychoactive substance that acts on various neurotransmitting mechanisms including gamma-aminobutyric acid, serotonin, glutamate, and opioid systems [26]. The substance is known to reduce stress and uplift one’s mood [27], and as emotion and pain are closely related [28], managing emotional states is known to achieve at least partial pain relief. However, a study on chronic pain through participation in an outpatient drug and alcohol treatment program reported that 29.1% of the alcoholic group reported severe chronic pain compared to 10% in the normal group [29], and in a 3-year study on an elderly population of individuals aged ≥62, problematic drinkers were more likely to suffer from severe pain which impeded their daily lives [30]. Moreover, recent reports have shown that radiological joint degeneration is not proportionate to the amount of pain [31, 32]. In light of the equivocal associations between symptomatic OA and radiological OA, and pain severity and drinking patterns, the relationship between pain severity and drinking pattern of knee OA patients is similarly hard put to be concluded.

Some strengths of this study include that participants were surveyed on a nationwide basis, and the interview was conducted in a systematic manner by experienced professionals. In addition, OA was diagnosed by physicians using KL grades based on X-ray findings, which is rare in national level studies such as this. Alcohol consumption was assessed using a validated and reliable method by means of the AUDIT questionnaire. Further, comprehensive adjustment of potential confounders, such as age, sex, income and education level, marital status, residential area, smoking status, BMI, and exercise, help increase the credibility of this study. In the current study, the three joints where OA is most commonly found – the knee, hip, and lumbar spine – were analyzed separately, and additional analysis was conducted for potential associations between pain severity and alcohol consumption. The greatest limitation of the current study probably lies in its cross-sectional design. As cross-sectional studies investigate associations assessed at a certain point of time, they are suitable for identifying associations, but not causal relationships. In addition, the small number of hip OA patients deterred proper conduction of statistical analysis, and no examinations other than radiological diagnosis were conducted for OA of the lumbar spine, rendering additional analyses on clinical symptoms and relevance for the lumbar spine impossible. The fact that the present study was conducted in a nationally representative population of Koreans may be viewed both as a strength and limitation, as the external reliability of the results is limited as a study performed within a homogeneous population. Lastly, the mechanism by which alcohol potentially increases risk of radiological knee OA is, as of yet, unclear, and the survey did not differentiate between different alcoholic beverages. Muthrui et al. suggested that beer, wine, and hard liquor may have different relationships with OA [33]. Additional analyses could not be conducted regarding type of alcoholic beverages in the present study due to such limitations of the raw data. Research on different types of alcoholic beverages and studies involving larger numbers of hip and lumbar spinal OA patients should be given due consideration.

## Conclusions

The current study found that higher AUDIT scores were significantly associated with increased risk of knee OA, but associations were not observed between AUDIT scores and pain severity. Further clinical research and well-designed prospective studies are warranted to clarify causal relationships and are hoped to aid the advancement of OA treatment and prevention.

## Data Availability

The datasets used and analyzed during the current study are available from the corresponding author on reasonable request. KNHANES data can be accessed and downloaded from the KNHANES website (https://knhanes.cdc.go.kr/knhanes/index.do).

## Abbreviations

AUDIT: Alcohol use disorders identification test
BMI: Body mass index
CI: Confidence interval
GDP: Gross domestic product
hsCRP: High-sensitivity C-reactive protein
KCDC: Korea Centers for Disease Control and Prevention
KL: Kellgren-Lawrence
KNHANES: Korean National Health and Nutrition Examination Survey
NRS: Numeric rating scale
OA: Osteoarthritis
OR: Odds ratio
SD: Standard deviation
